# Effects of Overexpression of the Egyptian Fruit Bat Innate Immune Genes on Filovirus Infections in the Host Cells

**DOI:** 10.3389/fviro.2021.759655

**Published:** 2021-10-12

**Authors:** Ivan V. Kuzmin, Palaniappan Ramanathan, Christopher F. Basler, Alexander Bukreyev

**Affiliations:** 1 Department of Pathology, University of Texas Medical Branch, Galveston, TX, United States; 2 Galveston National Laboratory, Galveston, TX, United States; 3 Center for Microbial Pathogenesis, Institute for Biomedical Sciences, Georgia State University, Atlanta, GA, United States; 4 Department of Microbiology and Immunology, University of Texas Medical Branch, Galveston, TX, United States

**Keywords:** BAT, innate immune response, innate immune system, Egyptian fruit bat, *Rousettus aegyptiacus*, Ebola virus, Marburg virus, filovirus

## Abstract

Bats constitute a large and diverse group of mammals with unique characteristics. One of these is the ability of bats to maintain various pathogens, particularly viruses, without evidence of disease. The innate immune system has been implicated as one of the important components involved in this process. However, in contrast to the human innate immune system, little data is available for bats. In the present study we generated 23 fusion constructs of innate immune genes of Egyptian fruit bat (*Rousettus aegyptiacus*) with mCherry as a fluorescent reporter. We evaluated the effects of overexpressing these genes on the replication of Marburg and Ebola viruses in the Egyptian fruit bat cell line R06EJ. Both viruses were substantially inhibited by overexpression of type I, II and III interferons, as well as by DDX58 (RIG-I), IFIH1, and IRF1. Our observations suggest that the broad antiviral activity of these genes reported previously in human cells is conserved in Egyptian fruit bats and these possess anti-filovirus activities that may contribute to the efficient virus clearance.

## INTRODUCTION

Marburg virus (MARV) and Ebola virus (EBOV), members of the family *Filoviridae,* cause severe disease with high case fatality rates in humans (1). Egyptian fruit bats (*Rousettus aegyptiacus*) have been identified as natural reservoir hosts of MARV (2–4). Several other divergent filoviruses were detected in bats of other species, including Lloviu virus (5), Reston virus (6), Bombali virus (7), Mengla virus (8). Although anecdotal findings of EBOV genetic material in bats was reported (9), this has not been corroborated despite the extensive surveillance. Egyptian fruit bats survive infection with MARV and EBOV without apparent clinical signs (4, 10, 11). Bats of other species exhibit subclinical infections and survive infections with henipaviruses and coronaviruses that cause severe diseases in humans (12–14). Many factors have been suggested to play a role in the resistance to disease in bats, with major attention given to their immune system and relatively reduced inflammatory responses (15–18).

Indeed, bats are very diverse with more than 1,400 species described, and the same great diversity might be expected to be seen in their adaptation to pathogens, many of which are species-specific. Nevertheless, the Egyptian fruit bat represents one of a few useful models for studying bat-pathogen interactions. These bats are easy to maintain and breed in captivity, they have relatively large body size (120–180 g) which makes them convenient for manipulations and for non-destructive sampling of excretions, cells, and tissues. Parts of the genome and transcriptome of the Egyptian fruit bat have been annotated (19–21), but more efforts are required to fully understand bat-pathogen interactions.

Among the components of mammalian immune system, type I interferon (IFN) response represent the first barrier to virus infection. Triggered by cellular pathogen-sensing receptors, it leads to activation of hundreds of so-called interferon-stimulated genes (ISGs) which engage in various mechanisms of virus elimination (22–24). Type II IFN is an important activator of macrophages and inducer of class I major histocompatibility complex (25, 26) which also plays a role in antiviral response, in part via cross-talk with the type I IFN pathway (26, 27). Type III IFNs are distributed unevenly in tissues and organs of different animals, and their roles in bat immunity have been studied minimally (28, 29). A few studies addressing antiviral response of Egyptian fruit bats to filoviruses provided diverse results (30–32) suggesting that more work in this area is required.

During the recent years, substantial efforts have been undertaken to decipher the role of human ISGs in response to different viruses via screening of large panels of ISGs overexpressed in human cells (33–36). In the present study we attempted to adapt one of these approaches to screening of a subset of IFNs and ISGs of the Egyptian fruit bat which demonstrated a differential expression in response to MARV and EBOV infections in our previous study (31).

## METHODS

### Cells and Viruses

Immortalized fetal cells of Egyptian fruit bats, R06EJ (37), kindly provided by Dr. Ingo Jordan (ProBioGen, Berlin, Germany) were maintained in DMEM F-12 GlutaMAX medium supplemented with 10% fetal bovine serum and antibiotic/antimycotic mix (Thermo Fisher Scientific).

The recombinant Marburg virus (MARV) of bat origin (38) expressing enhanced green fluorescent protein (eGFP) was recovered using the MARV reverse genetics systems provided by Drs. Jonathan Towner, Cesar Albarino, and Stuart Nichol (CDC). The recombinant Ebola virus (EBOV) strain Mayinga (39) expressing eGFP was recovered using the EBOV reverse genetics system including the full-length clone provided by Drs. Jonathan Towner and Stuart Nichol and the EBOV NP, VP35, L, VP30, and T7 polymerase plasmids provided by Drs. Yoshihiro Kawaoka (University of Wisconsin) and Heinz Feldmann (NIH). Both viruses were passaged 3 times in Vero E6 cells.

### Construction of Expression Vectors for Bat Innate Immune Genes

The mRNA sequences of the selected genes were retrieved from GenBank and amplified via nested RT-PCR from R06EJ cells or from liver and spleen tissues of healthy Egyptian fruit bats, with primer-derived removal of stop codons and *ad hoc* addition of restriction sites. The amplicons were inserted under control of the chicken β-actin promoter into pCAGGS/MCS vector expressing mCherry with flexible linker upstream (2 × ggtggcggaggtggctca), so that mCherry was expressed in-frame with the inserted bat genes. The insertions were created either using NotI and EcoRV restriction sites or, if these sites were present in the bat genes, via seamless ligation into blunt-ended backbone using the GeneArt Seamless Cloning and Assembly kit (Thermo Fisher Scientific). For each construct, 5–10 colonies were selected for sequence verification, and clones with correct sequences were scaled-up for further studies. The empty pCAGGS/MCS vector expressing mCherry connected to the flexible linker was used as negative (mock) control.

### Transfection, Infection, and Flow Cytometry

**FIGURE 1 |:**
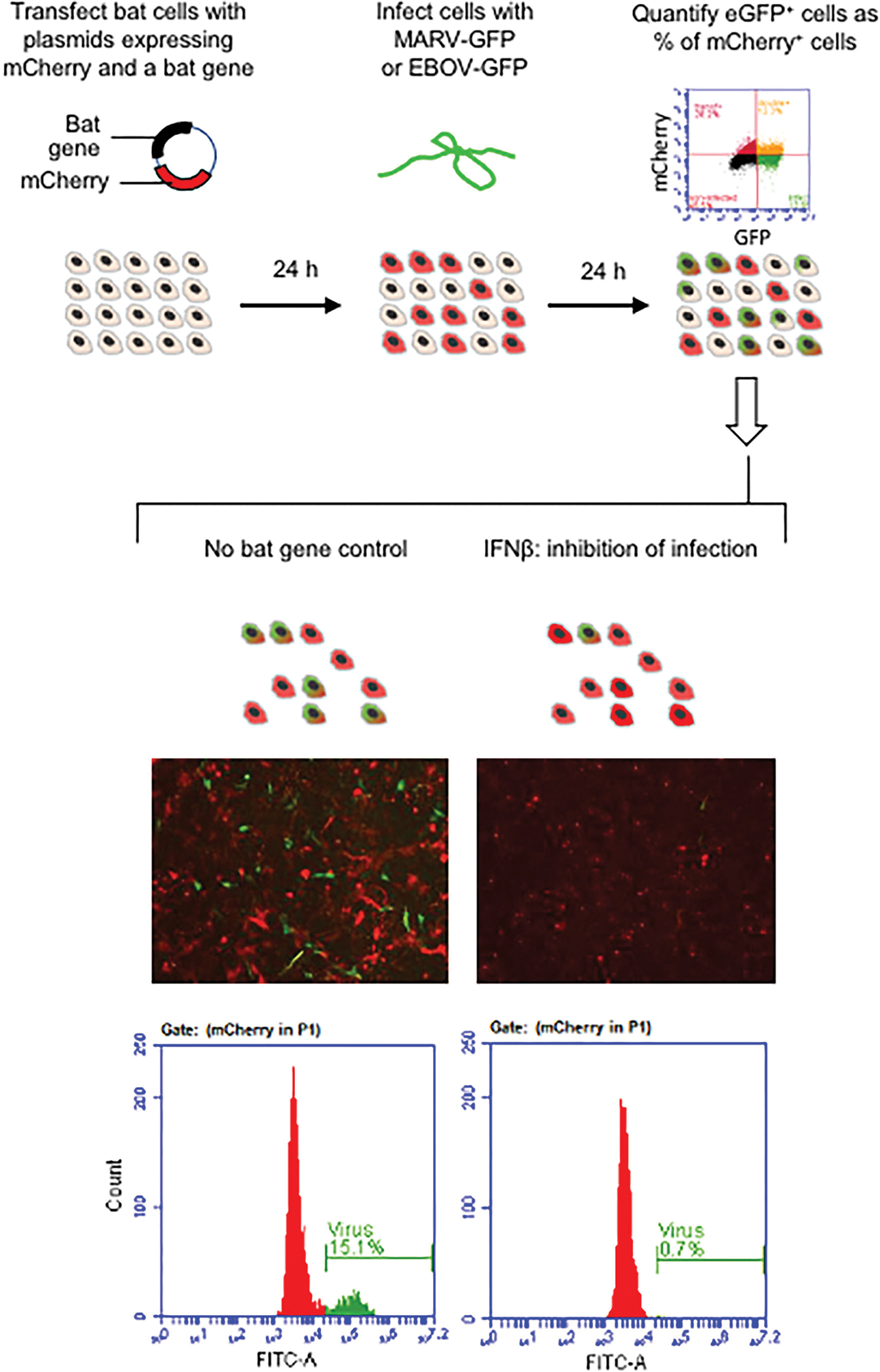
Schematic illustration of the experimental workflow. The microphotographs show cells transfected by empty mCherry^+^ plasmid (mock) and inhibitory IFNβ following infection with MARV-GFP.

The experimental approach was similar to that described previously (35, 36). Expression plasmids were transfected into subconfluent R06EJ cells in 6-well plates using TransIT-X2 reagent (Mirus Bio LLC, Madison, WI) following manufacturers instructions. Each construct was tested in triplicates. Twenty-four h post transfection, the cells were washed with phosphate buffered saline (PBS) and infected with MARV (MOI of 1 PFU/cell, as determined in Vero E6 cells) or EBOV (MOI of 3 PFU/cell, as determined in Vero E6 cells) in the BSL-4 containment of the Galveston National Laboratory. After adsorption for one h at 37 °C, the cells were washed twice with PBS, and supplied with fresh medium. Twenty-four h post infection, the cells were removed from plates with trypsin-EDTA, washed in PBS, fixed in 4% paraformaldehyde, and subjected to flow cytometry on an Accuri C6 (BD Biosciences). From each well, 100,000–150,000 cells were counted. The results were analyzed in C6 Plus Analysis software (BD Biosciences) with a 0.1% compensation. Only mCherry^+^ cells were selected for comparative evaluation of the proportions of eGFP^+^ cells (Figure 1). Whenever transfection of bat genes demonstrated an increase or decrease of infected cells compared to mock-transfected control, the experiments were repeated for corroboration of results. Statistical differences between sample triplicates were assessed by Students two-tailed unpaired *t*-test. Significance levels were set at *p* ≤ 0.05.

## RESULTS

### Selection, Sequencing and Cloning of Egyptian Fruit Bat Innate Immune Genes

We selected a subset of innate immune genes of the Egyptian fruit bat that demonstrated the most distinct expression patterns in R06EJ cells infected with MARV and EBOV, compared to noninfected cells, in our previous study (31), Table 1. To test the antiviral effect of the genes, we attempted to adapt the system developed previously for screening of the antiviral effects of human genes in human cells (35, 36). The system we developed was based on transfection of cells with plasmids expressing bat genes of interest and a fluorescent protein mCherry, and infection of bat cells with MARV or EBOV engineered to express another fluorescent protein, eGFP (38, 39) (Figure 1, top). The levels of antiviral activities were determined by flow cytometry analysis of cells and calculation of the percentage of eGFP^+^ (infected) cells as a percentage of mCherry^+^ (plasmid-transfected) cells.

The empty pCAGGS/MCS vector expressing mCherry connected to the flexible linker was used as negative (mock) control. After RT-PCR amplification of bat genes, we compared all our sequences to those deposited in GenBank, and did not have discrepancies in most cases. One exception was the sequence of IFNα which was selected among >20 diverse clones obtained from single RT-PCR amplification. This is not surprising given that 46 type I IFNs, including 12 IFNα variants, were identified in the Egyptian fruit bat genome (20). We selected the sequence from the clone that most closely resembled the consensus IFNα sequence obtained via Sanger sequencing of the RT-PCR product, assuming that this variant predominates in the IFNα population. Another interesting finding was the IFIT3 sequence contained a stop codon within the expected coding region (amino acid position 346). It was present in the RT-PCR product and in all otherwise identical clones. Upon inspection of GenBank record XM_016123309, we found the same stop codon and concluded that CDS was determined and annotated in GenBank incorrectly. Therefore, we re-amplified and used in our studies a truncated version of IFIT3 consisting of 345 codons. We were unable to amplify two genes of interest, IFITM3 and IRF7, either from bat cells or organs, likely because of limited representation of the mRNA in the samples.

### Establishing the System for Screening of Bat Innate Immune Genes for the Antiviral Effect in Bat Cells

To screen for the antiviral activity, we selected R06EJ cells because they are derived from Egyptian fruit bat, which is the natural reservoir for MARV (4), because they were more susceptible to infection with EBOV compared to another available cell line derived from the Egyptian fruit bat, RoNi/7, and because they were better transfected in our previous study (31). As was observed previously by other investigators (34, 36), overexpression of various immune genes was toxic to cells, and that was the reason why we performed the study with large numbers of cells in 6-well plates (approximately 1,300,000 R06EJ cells per well), given that over 50% of cells might be lost after transfection. Nevertheless, we had to remove from evaluation the TNFSF10 construct which was so toxic that only singular mCherry^+^ cells were attached to the plate 24 h after transfection. The selected transfection reagent TransIT-X2 did not demonstrate severe cytotoxicity to R06EJ cells and was reasonably efficient such that 15–30% cells were mCherry^+^ when the empty mCherry vector was used. The cells transfected with this vector demonstrated healthy morphology, whereas the cells transfected with most of the bat immune genes appeared rounded and in smaller sizes (Figure 1) even if they were firmly attached to the plates during all manipulations and the following observation period.

Another limiting factor of our study was the susceptibility of cells to EBOV. While the Egyptian fruit bat is the natural host of MARV, it is almost refractory to EBOV (4, 11). In fact, R06EJ cells were more susceptible to MARV (up to 15% cells were eGFP^+^ after infection at MOI of 1 PFU/cell) than to EBOV (about 10% cells were eGFP^+^ after infection at MOI of 3 PFU/cell). With all these limitations, even using the 6-well format and counting 100,000–1,500,000 cells per well at flow cytometry, we were able to detect ~1,000–5,000 mCherry^+^, eGFP^+^ cells in mock-control wells (which were considered as 100% infection in our quantitation). After optimization of the system, we chose the experimental layout which included plasmid transfection, followed by a 24 h-long incubation, infection, a 24 h-long incubation, and flow cytometry analysis (Figure 1).

### The Impact of Overexpressed Bat Genes Upon Filovirus Infection

**FIGURE 2 |:**
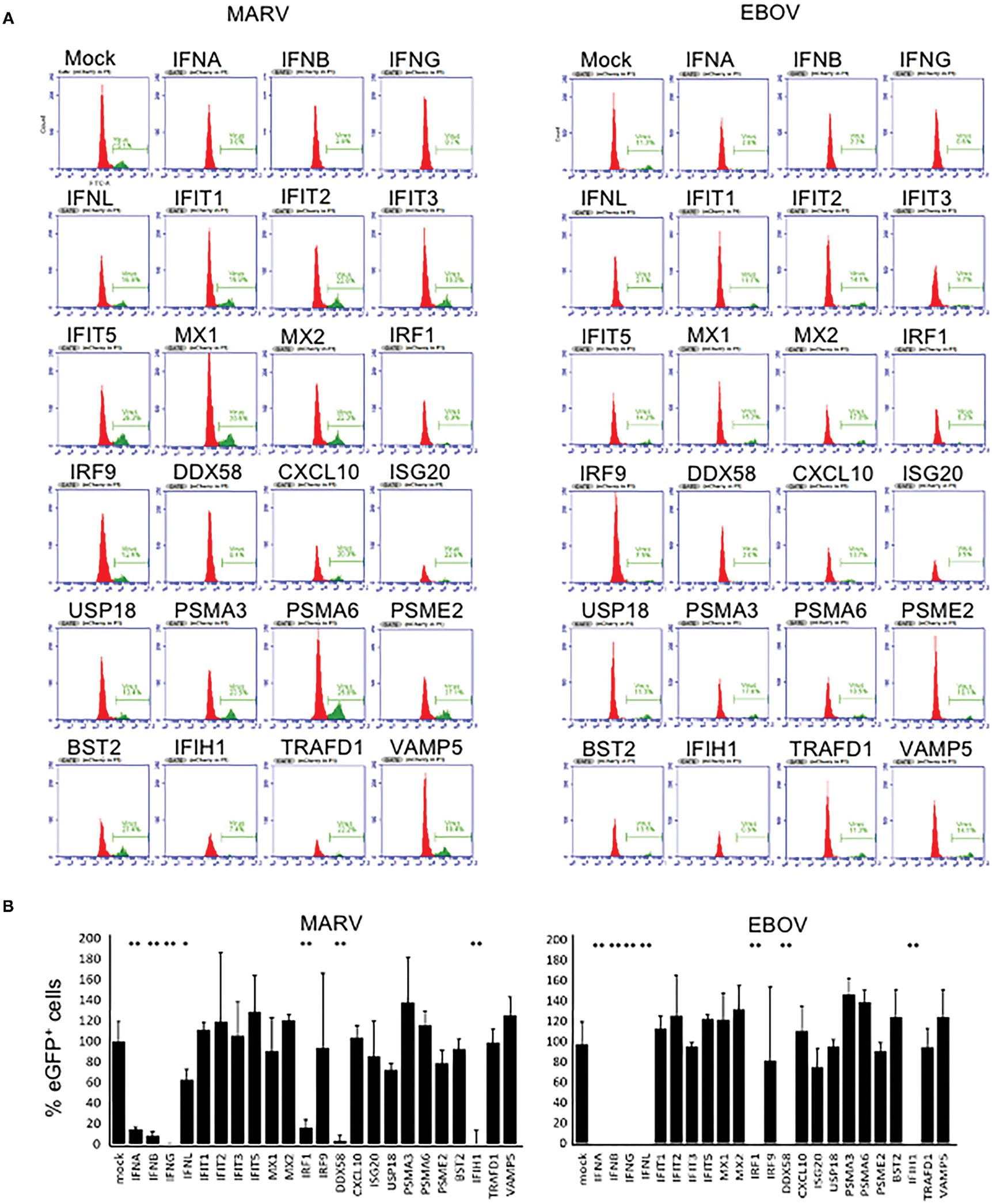
Antiviral effects of bat immune genes. **(A)** Flow cytometry histograms showing percentages of eGFP^+^ (infected) cells among mCherry^+^ (plasmid transfected) cells. **(B)** Percentages of infected (eGFP^+^) cells among the cells transfected with bat immune genes (mCherry^+^). The 100% value corresponds to the proportion of eGFP^+^ cells in control (mock; mCherry^+^ plasmid with no immune gene). Means of triplicate samples ± SD. Asterisks indicate statistical significance at 95% (*) and 99% (**). The study was performed two times which results in similar data.

The effects of overexpressed bat genes to MARV and EBOV were similar (Figure 2). In the cells transfected with plasmids expressing type I and type II IFNs, virus replication (eGFP signal) was observed only in a small numbers of cells (Figures 1, 2). This was true not only for the mCherry^+^ cells but for all cells in the sample, in agreement with IFN excretion that affects bystander cells as was documented elsewhere (22–24, 26) and in our previous study (31). The type III IFN (IFNλ) had a lesser effect on the proportion of eGFP^+^ cells infected with MARV (50–70% reduction) but effectively suppressed replication of EBOV for over 90% (Figure 2). Overexpression of three other genes, IRF1, DDX58 (RIG-I) and IFIH1 inhibited MARV and EBOV in the transfected cells (Figure 2). Other genes overexpressed in our experiments had either no effect to the proportion of eGFP^+^ cells or slightly increased this proportion (IFIT1 by 6–21%; IFIT5 by 22–53%; MX2 by 11–33%; PSMA3 by 14–46; PSMA6 by 8–36%, VAMP5 by 7–34%).

## DISCUSSION

The bat immune system attracts increasing scientific attention due to the ability of bats to maintain and clear various viral infections without apparent clinical signs (20, 32). Innate immune response may be an important component of such effective tolerance of pathogens.

Several large-scale studies of human innate immune genes transiently expressed in human cells and their effects to various pathogens have been published in recent years (33–36, 40). However, no such studies were published for bat immune genes at the time of submission of this manuscript. Here we amplified and sequenced 23 innate immune genes of Egyptian fruit bats, cloned them in expression plasmids, and developed a system for evaluation of their antiviral effect against MARV and EBOV in bat cells. While bat immune system shares many key features with humans and other mammals, bats also possess so-called “unique” immune characteristics and functional differences in the regulation of their innate immune systems (28, 41–43). Therefore, direct assessment of antiviral effects of bat immune genes are of immediate importance.

We observed strong antiviral effects of overexpressed and secreted IFNs type I, II, and III against MARV and EBOV. We used fusion constructs of IFNs with mCherry, and if they are secreted, we would not observe mCherry^+^ cells. The observed presence of mCherry^+^ cells can be related to ongoing synthesis of the proteins from the transfected plasmids. Alternatively, the level of secretion may be reduced due to the fused mCherry, or mCherry is released from dead cells. The IFN effects resembled those described in human cells and are typical for mammals in general (26, 44). We overexpressed one of the 12 IFNα variants detected in the genome of Egyptian fruit bat (20). As the genome of this bat demonstrates the presence of 46 type I IFN genes (20), additional studies are required to address the effects of each of them against filoviruses and other pathogens. The type III IFN demonstrated a lesser activity against MARV than type I and type II IFNs. Type III IFNs are represented by several species which have not been annotated and characterized in the Egyptian fruit bat yet (32), and each may have its unique role in suppression of filoviruses and other pathogens. An antiviral effect of IFNλ was demonstrated, for example, against Pulau virus (family *Reoviridae*) in *Pteropus* cells (28, 29).

A broad activity of human IRF1, DDX58 (RIG-I) and IFIH1 against diverse viruses, including EBOV, was demonstrated previously in human cells (34, 36). Also, the same authors did not see substantial antiviral effects from overexpression of ISGs IFIT1–5 and MX1–2, although these genes are highly expressed in infected cells, including bat cells R06EJ (31). It is likely that these and the majority of other ISGs play their roles in the innate immune pathways but do not possess direct antiviral activity.

Interestingly, despite the very different susceptibility of Egyptian fruit bats to MARV and EBOV (4) and different mechanisms of antagonism of IFN induction and response by these viruses (45), comparison of the antiviral effects of the 23 bat immune genes demonstrated very similar profiles with equally strong activity for six of them against the two viruses.

A limitation of our study was that innate immune genes were expressed as fusion proteins, which might not function, localize, or be secreted properly. The design of our experiments did not allow us to distinguish whether overexpressed bat genes influenced virus entry or replication. Furthermore, as the observations were limited to 24 hours, and supernatants of the cultures were not tested, we did not address whether the overexpressed genes affected virus budding. That was a limitation highlighted, for example, by observations of other researchers that BST2 specifically inhibits virus budding from human cells (34, 46). In our case, BST2 did not reduce the number of eGFP^+^ cells, however, we do not know whether the viruses were budding efficiently and what were their concentrations in the supernatants.

Further studies are required to more completely assess the bat innate immune system. Identification and characterization of additional bat immune genes is essential. For example, our work identified an unexpected stop codon within the proposed IFIT3 coding sequence, and some mRNAs are difficult to amplify via conventional RT-PCR which possibly suggests a polymorphism. With more data and reagents available, biological effects of bat immune system against a broad range of pathogens, and the precise role in bat IFN signaling cascades should be elucidated. Ultimately, all these studies will contribute to our understating of the protective mechanisms which allow bats to effectively control replication of many highly pathogenic viruses without an acute disease.

## Figures and Tables

**TABLE 1 |:** Bat innate immune genes transiently overexpressed in the present study.

Protein	Gene/protein abbreviation	Functions (abbreviated)

Interferon alpha	IFNA, IFNα	Type I interferon, secreted in response to viral infection and mediates innate immune response by induction of multiple interferon-stimulated genes (ISGs) aimed to inhibit the infection by various means.
Interferon beta	IFNB, IFNβ	The same.
Interferon gamma	IFNG, IFNγ	Type II interferon, primarily secreted by specialized immune cells, promotes macrophage activation, enhances antigen presentation, coordinates lymphocyte-endothelium interaction, regulates Th1/Th2 balance, in addition, stimulates type I interferon response via circular loop.
Interferon lambda	IFNL, IFNλ	Type III interferon. Induces expression of ISGs and exert antiviral properties in a similar manner to type I IFNs. In addition to ubiquitous functioning promotes antiviral response in barrier areas such as respiratory epithelium and blood-brain barrier.
Interferon-induced protein with tetratricopeptide repeats 1	IFIT1	Specifically binds single-stranded RNA, thereby acting as a sensor of viral single-stranded RNAs and inhibiting expression of viral messenger RNAs.
Interferon-induced protein with tetratricopeptide repeats 2	IFIT2	Inhibits expression of viral messenger RNAs. Can promote apoptosis.
Interferon-induced protein with tetratricopeptide repeats 3	IFIT3	Enhances host antiviral responses by serving as an adapter bridging TBK1 to MAVS which leads to the activation of TBK1 and phosphorylation of IRF3 which translocates into nucleus to promote antiviral gene transcription.
Interferon-induced protein with tetratricopeptide repeats 5	IFIT5	Binds single-stranded RNA, thereby acting as a sensor and inhibitor of viral single-stranded RNAs.
Interferon-induced GTP-binding protein Mx1	MX1	Dynamin-like GTPase, binds and inactivates viral ribonucleocapsid.
Interferon-induced GTP-binding protein Mx2	MX2	The same, may play a role in regulating nucleocytoplasmic transport and cell-cycle progression.
Interferon regulatory factor 1	IRF1	Regulates transcription of type I interferons and ISGs in host response to viral and bacterial infections.
Interferon regulatory factor 9	IRF9	Mediates signaling by type I interferons during viral infection.
Antiviral innate immune response receptor RIG-I	DDX58	Senses cytoplasmic viral nucleic acids and activates a downstream signaling cascade leading to the production of type I interferons and proinflammatory cytokines.
C-X-C motif chemokine 10	CXCL10	Involved in a wide variety of processes such as chemotaxis, differentiation, and activation of peripheral immune cells, regulation of cell growth, apoptosis.
Interferon-stimulated gene 20 kDa protein	ISG20	An exoribonuclease that acts on single-stranded RNA and also has minor activity toward single-stranded DNA.
Ubl carboxyl-terminal hydrolase 18	USP18	Involved in modulation of the inflammatory response triggered by type I interferons.
Proteasome subunit alpha type 3	PSMA3	Component of the 20S core proteasome complex involved in the proteolytic degradation of most intracellular proteins. During viral infections mediates apoptosis.
Proteasome subunit alpha type 6	PSMA6	The same.
Proteasome activator complex subunit 2	PSME2	Implicated in immunoproteasome assembly and required for efficient antigen processing.
Bone marrow stromal antigen 2	BST2	Blocks the release of diverse mammalian enveloped viruses by directly tethering nascent virions to the membranes of infected cells. Acts as a direct physical tether, holding virions to the cell membrane and linking virions to each other.
Interferon-induced helicase C domain-containing protein 1	IFIH1	Sensor of viral nucleic acids and plays a major role in sensing viral infection and in the activation of a cascade of antiviral responses including the induction of type I interferons and proinflammatory cytokines.
TRAF-type zinc finger domain-containing protein 1	TRAFD1	Negative feedback regulator that controls excessive innate immune responses mediated by Toll-like receptor 4 and DDX58/RIG1-like pathways.
Vesicle-associated membrane protein 5	VAMP5	Intracellular trafficking events.

## Data Availability

The raw data supporting the conclusions of this article will be made available by the authors, without undue reservation.

## References

[1] Kuhn JH, Amarasinghe GK, Perry DL. Filoviridae: Marburg and Ebola Viruses. (Lippincott-Raven Publishers: Philadelphia). (2021).

[2] Towner JS, Amman BR, Sealy TK, Carroll SA, Comer JA, Kemp A, Isolation of genetically diverse Marburg viruses from Egyptian fruit bats. PLoS Pathog. (2009) 5:e1000536. doi: 10.1371/journal.ppat.1000536 19649327 PMC2713404

[3] Amman BR, Bird BH, Bakarr IA, Bangura J, Schuh AJ, Johnny J, Isolation of Angola-like Marburg virus from Egyptian rousette bats from West Africa. Nat Commun. (2020) 11:510. doi: 10.1038/s41467-020-14327-8 31980636 PMC6981187

[4] Jones ME, Schuh AJ, Amman BR, Sealy TK, Zaki SR, Nichol ST, Experimental inoculation of egyptian rousette bats (rousettus aegyptiacus) with viruses of the ebolavirus and marburgvirus genera. Viruses. (2015) 7:3420–42. doi: 10.3390/v7072779 26120867 PMC4517108

[5] Negredo A, Palacios G, Vázquez-Morón S, González F, Dopazo H, Molero F, Discovery of an ebolavirus-like filovirus in europe. PLoS Pathog. (2011) 7:e1002304. doi: 10.1371/journal.ppat.1002304 22039362 PMC3197594

[6] Jayme SI, Field HE, de Jong C, Olival KJ, Marsh G, Tagtag AM, Molecular evidence of Ebola Reston virus infection in Philippine bats. Virol J. (2015) 12:107. doi: 10.1186/s12985-015-0331-3 26184657 PMC4504098

[7] Goldstein T, Anthony SJ, Gbakima A, Bird BH, Bangura J, Tremeau-Bravard A, The discovery of Bombali virus adds further support for bats as hosts of ebolaviruses. Nat Microbiol. (2018) 3:1084–9. doi: 10.1038/s41564-018-0227-2 30150734 PMC6557442

[8] Yang XL, Tan CW, Anderson DE, Jiang RD, Li B, Zhang W, Characterization of a filovirus (Měnglà virus) from Rousettus bats in China. Nat Microbiol. (2019) 4:390–5. doi: 10.1038/s41564-018-0328-y 30617348

[9] Leroy EM, Kumulungui B, Pourrut X, Rouquet P, Hassanin A, Yaba P, Fruit bats as reservoirs of Ebola virus. Nature. (2005) 438:575–6. doi: 10.1038/438575a 16319873

[10] Paweska JT, Jansen van Vuren P, Masumu J, Leman PA, Grobbelaar AA, Birkhead M, Virological and serological findings in Rousettus aegyptiacus experimentally inoculated with vero cells-adapted hogan strain of Marburg virus. PLoS ONE. (2012) 7:e45479. doi: 10.1371/journal.pone.0045479 23029039 PMC3444458

[11] Paweska JT, Storm N, Grobbelaar AA, Markotter W, Kemp A, Jansen van Vuren P. Experimental inoculation of egyptian fruit bats (rousettus aegyptiacus) with ebola virus. Viruses. (2016) 8:29. doi: 10.3390/v8020029 PMC4776184 26805873

[12] Halpin K, Hyatt AD, Fogarty R, Middleton D, Bingham J, Epstein JH, Pteropid bats are confirmed as the reservoir hosts of henipaviruses: a comprehensive experimental study of virus transmission. Am J Trop Med Hyg. (2011) 85:946–51. doi: 10.4269/ajtmh.2011.10-0567 22049055 PMC3205647

[13] Middleton DJ, Morrissy CJ, van der Heide BM, Russell GM, Braun MA, Westbury HA, Experimental Nipah virus infection in pteropid bats (Pteropus poliocephalus). J Comp Pathol. (2007) 136:266–72. doi: 10.1016/j.jcpa.2007.03.002 17498518

[14] Schlottau K, Rissmann M, Graaf A, Schön J, Sehl J, Wylezich C, SARS-CoV-2 in fruit bats, ferrets, pigs, and chickens: an experimental transmission study. Lancet Microbe. (2020) 1:e218–e25. doi: 10.1016/S2666-5247(20)30089-6 32838346 PMC7340389

[15] Guito JC, Prescott JB, Arnold CE, Amman BR, Schuh AJ, Spengler JR, Asymptomatic infection of marburg virus reservoir bats is explained by a strategy of immunoprotective disease tolerance. Curr Biol. (2021) 31:257–70.e5. doi: 10.1016/j.cub.2020.10.015 33157026

[16] Prescott J, Guito JC, Spengler JR, Arnold CE, Schuh AJ, Amman BR, Rousette bat dendritic cells overcome marburg virus-mediated antiviral responses by upregulation of interferon-related genes while downregulating proinflammatory disease mediators. mSphere. (2019) 4:e00728–19. doi: 10.1128/mSphere.00728-19 31801842 PMC6893212

[17] Clayton E, Munir M. Fundamental characteristics of bat interferon systems. Front Cell Infect Microbiol. (2020) 10:527921. doi: 10.3389/fcimb.2020.527921 33363045 PMC7759481

[18] Irving AT, Zhang Q, Kong PS, Luko K, Rozario P, Wen M, Interferon regulatory factors IRF1 and IRF7 directly regulate gene expression in bats in response to viral infection. Cell Rep. (2020) 33:108345. doi: 10.1016/j.celrep.2020.108345 33147460 PMC8755441

[19] Lee AK, Kulcsar KA, Elliott O, Khiabanian H, Nagle ER, Jones ME, De novo transcriptome reconstruction and annotation of the Egyptian rousette bat. BMC Genomics. (2015) 16:1033. doi: 10.1186/s12864-015-2124-x 26643810 PMC4672546

[20] Pavlovich SS, Lovett SP, Koroleva G, Guito JC, Arnold CE, Nagle ER, The egyptian rousette genome reveals unexpected features of bat antiviral immunity. Cell. 173:1098–110.e18. doi: 10.1016/j.cell.2018.03.070 PMC7112298 29706541

[21] Jebb D, Huang Z, Pippel M, Hughes GM, Lavrichenko K, Devanna P, Six reference-quality genomes reveal evolution of bat adaptations. Nature. (2020) 583:578–84. doi: 10.1038/s41586-020-2486-3 32699395 PMC8075899

[22] Pestka S, Krause CD, Walter MR. Interferons, interferon-like cytokines, their receptors. Immunol Rev. (2004) 202:8–32. doi: 10.1111/j.0105-2896.2004.00204.x 15546383

[23] Takaoka A, Yanai H. Interferon signalling network in innate defence. Cell Microbiol. (2006) 8:907–22. doi: 10.1111/j.1462-5822.2006.00716.x 16681834

[24] Yang E, Li MMH. All about the RNA: interferon-stimulated genes that interfere with viral RNA processes. Front Immunol. (2020) 11:605024. doi: 10.3389/fimmu.2020.605024 33362792 PMC7756014

[25] Ben-Asouli Y, Banai Y, Pel-Or Y, Shir A, Kaempfer R. Human interferon-gamma mRNA autoregulates its translation through a pseudoknot that activates the interferon-inducible protein kinase PKR. Cell. (2002) 108:221–32. doi: 10.1016/S0092-8674(02)00616-5 11832212

[26] Rhein BA, Powers LS, Rogers K, Anantpadma M, Singh BK, Sakurai Y, Interferon-γ Inhibits Ebola Virus Infection. PLoS Pathog. (2015) 11:e1005263. doi: 10.1371/journal.ppat.1005263 26562011 PMC4643030

[27] Liu SY, Sanchez DJ, Aliyari R, Lu S, Cheng G. Systematic identification of type I and type II interferon-induced antiviral factors. Proc Natl Acad Sci U S A. (2012) 109:4239–44. doi: 10.1073/pnas.1114981109 22371602 PMC3306696

[28] Zhou P, Cowled C, Marsh GA, Shi Z, Wang LF, Baker ML. Type III IFN receptor expression and functional characterisation in the pteropid bat, Pteropus alecto. PLoS ONE. (2011) 6:e25385. doi: 10.1371/journal.pone.0025385 21980438 PMC3181264

[29] Zhou P, Cowled C, Todd S, Crameri G, Virtue ER, Marsh GA, Type III IFNs in pteropid bats: differential expression patterns provide evidence for distinct roles in antiviral immunity. J Immunol. (2011) 186:3138–47. doi: 10.4049/jimmunol.1003115 21278349 PMC3057921

[30] Hölzer M, Krähling V, Amman F, Barth E, Bernhart SH, Carmelo VA, Differential transcriptional responses to Ebola and Marburg virus infection in bat and human cells. Sci Rep. (2016) 6:34589. doi: 10.1038/srep34589 27713552 PMC5054393

[31] Kuzmin IV, Schwarz TM, Ilinykh PA, Jordan I, Ksiazek TG, Sachidanandam R, Innate immune responses of bat and human cells to filoviruses: commonalities and distinctions. J Virol. (2017) 91:e02471–16. doi: 10.1128/JVI.02471-16 28122983 PMC5375674

[32] Arnold CE, Guito JC, Altamura LA, Lovett SP, Nagle ER, Palacios GF, Transcriptomics reveal antiviral gene induction in the egyptian rousette bat is antagonized *in vitro* by marburg virus infection. Viruses. (2018) 10:607. doi: 10.3390/v10110607 PMC6266330 30400182

[33] Kane M, Zang TM, Rihn SJ, Zhang F, Kueck T, Alim M, Identification of interferon-stimulated genes with antiretroviral activity. Cell Host Microbe. (2016) 20:392–405. doi: 10.1016/j.chom.2016.08.005 27631702 PMC5026698

[34] Kuroda M, Halfmann PJ, Hill-Batorski L, Ozawa M, Lopes TJS, Neumann G, Identification of interferon-stimulated genes that attenuate Ebola virus infection. Nat Commun. (2020) 11:2953. doi: 10.1038/s41467-020-16768-7 32528005 PMC7289892

[35] Schoggins JW, MacDuff DA, Imanaka N, Gainey MD, Shrestha B, Eitson JL, Pan-viral specificity of IFN-induced genes reveals new roles for cGAS in innate immunity. Nature. (2014) 505:691–5. doi: 10.1038/nature12862 24284630 PMC4077721

[36] Schoggins JW, Wilson SJ, Panis M, Murphy MY, Jones CT, Bieniasz P, A diverse range of gene products are effectors of the type I interferon antiviral response. Nature. (2011) 472:481–5. doi: 10.1038/nature09907 21478870 PMC3409588

[37] Jordan I, Horn D, Oehmke S, Leendertz FH, Sandig V. Cell lines from the Egyptian fruit bat are permissive for modified vaccinia Ankara. Virus Res. (2009) 145:54–62. doi: 10.1016/j.virusres.2009.06.007 19540275 PMC7172177

[38] Albariño CG, Wiggleton Guerrero L, Spengler JR, Uebelhoer LS, Chakrabarti AK, Nichol ST, Recombinant Marburg viruses containing mutations in the IID region of VP35 prevent inhibition of Host immune responses. Virology. (2015) 476:85–91. doi: 10.1016/j.virol.2014.12.002 25531184 PMC6461211

[39] Towner JS, Paragas J, Dover JE, Gupta M, Goldsmith CS, Huggins JW, Generation of eGFP expressing recombinant Zaire ebolavirus for analysis of early pathogenesis events and high-throughput antiviral drug screening. Virology. (2005) 332:20–7. doi: 10.1016/j.virol.2004.10.048 15661137

[40] Li J, Ding SC, Cho H, Chung BC, Gale M Jr, Chanda SK, A short hairpin RNA screen of interferon-stimulated genes identifies a novel negative regulator of the cellular antiviral response. MBio. (2013) 4:e00385–13. doi: 10.1128/mBio.00385-13 23781071 PMC3684836

[41] De La Cruz-Rivera PC, Kanchwala M, Liang H, Kumar A, Wang LF, Xing C, The IFN Response in Bats Displays Distinctive IFN-Stimulated Gene Expression Kinetics with Atypical RNASEL Induction. J Immunol. (2018) 200:209–17. doi: 10.4049/jimmunol.1701214 29180486 PMC5736455

[42] Zhou P, Tachedjian M, Wynne JW, Boyd V, Cui J, Smith I, Contraction of the type I IFN locus and unusual constitutive expression of IFN-α in bats. Proc Natl Acad Sci U S A. (2016) 113:2696–701. doi: 10.1073/pnas.1518240113 26903655 PMC4790985

[43] Baker ML, Schountz T, Wang LF. Antiviral immune responses of bats: a review. Zoonoses Public Health. (2013) 60:104–16. doi: 10.1111/j.1863-2378.2012.01528.x 23302292 PMC7165715

[44] Stetson DB, Medzhitov R. Type I interferons in host defense. Immunity. (2006) 25:373–81. doi: 10.1016/j.immuni.2006.08.007 16979569

[45] Messaoudi I, Amarasinghe GK, Basler CF. Filovirus pathogenesis and immune evasion: insights from Ebola virus and Marburg virus. Nat Rev Microbiol. (2015) 13:663–76. doi: 10.1038/nrmicro3524 26439085 PMC5201123

[46] Kaletsky RL, Francica JR, Agrawal-Gamse C, Bates P. Tetherin-mediated restriction of filovirus budding is antagonized by the Ebola glycoprotein. Proc Natl Acad Sci U S A. (2009) 106:2886–91. doi: 10.1073/pnas.0811014106 19179289 PMC2650360

